# An interpretive phenomenological analysis of formative feedback in anesthesia training: the residents’ perspective

**DOI:** 10.1186/s12909-020-02402-z

**Published:** 2020-12-07

**Authors:** Krista C. Ritchie, Ana Sjaus, Allana Munro, Ronald B. George

**Affiliations:** 1 Faculty of Education, Mount Saint Vincent University, 166 Bedford Highway, Halifax, NS B3M2J6 Canada; 2 Department of Anesthesiology, Pain Management and Perioperative Medicine, Dalhousie University, Halifax, NS Canada; 3 IWK Health Centre, Halifax, NS Canada; 4 University of California San Francisco, San Francisco, CA USA

**Keywords:** Resident feedback, Clinical settings, Interpretive phenomenology, Assessment for learning, Feedback, Competence-by-design

## Abstract

**Background:**

Consistent formative feedback is cornerstone to competency-by-design programs and evidence-based approaches to teaching and learning processes. There has been no published research investigating feedback from residents’ perspectives. We explored the value residents place on feedback in routine operating room settings, their experiences, and understanding of the role of feedback in their training and developing professional identity.

**Methods:**

Interpretive phenomenological analysis of residents’ experiences with feedback received in clinical settings involved two focus groups with 14 anesthesia residents at two time points. Analysis was completed in the context of a teaching hospital adapting to new practices to align with nationally mandated clinical competencies. Focus group conversations were transcribed and interpreted through the lens of a social constructivist approach to learning as a dynamic inter- and intra-personal process, and evidence-based assessment standards set by the International Test Commission (ITC).

**Results:**

Residents described high quality feedback as consistent, effortful, understanding of residents’ thought processes, and containing actionable advice for improvement. These qualities of effective evaluation were equally imperative for informal and formal evaluations. Residents commented that highest quality feedback was received informally, and formal evaluations often lacked what they needed for their professional development.

**Conclusion:**

Residents have a deep sense of what promotes their learning. Structured feedback tools were seen positively, although the most important determinants of their impact were faculty feedback- and broader evaluation-skills and motivations for both formal and informal feedback loops.

## Background

With the advent of the Royal College of Physicians and Surgeons of Canada (RCPSC) competence by design (CBD) residency programs in Canada [1], in response to a desired competency-based medical education (CBME), there is renewed debate regarding how to develop and assess competence in residency programs. CBME has adopted a milestone oriented assessment structure that involves a clear learning path, frequent observations in practice settings, meaningful feedback, time and opportunity to develop new skills, and committee assessment of readiness to iteratively progress and professionally practice at appropriate levels [2]. This renewed language around resident learning and assessment gives residency training programs the opportunity to reflect on and adapt current practices in support of optimal resident learning. This study stemmed from an accredited Canadian residency program on the path of implementing evidence-based education to achieve the CBME guidelines.

Assessment, evaluation and feedback are, in medicine, often used interchangeably and with significant overlap, reflective of the relative lack of definitions specific to the context. Assessment is an estimation of the nature, quality, or ability of someone or something. An assessment of a learner involves gathering evidence about the learner’s performance while evaluation measures performance against desired educational outcomes. Feedback is the method used to inform the learner or other stakeholder of the result in fulfilment of specific educational goals [3]. Assessment that is accurate and relevant to a learner who is responsible for demonstrating competence will be most useful to further engage and propel them forward. This is the expectation set for residents by the RCPSC [1]. Given the socially situated, complex and highly interpersonal nature of clinical skills, longitudinal assessment of a specific person on a specific skill is a difficult task. Evaluation is formative in nature when accurate evidence about resident clinical competence is elicited, interpreted, and used to make decisions about the next steps in learning. These decisions are then likely to be better, or better founded, than the decisions they would have made in the absence of elicited evidence [4]. This formative and situated approach to evaluation results in feedback that is categorically different from global rating scales of overall quality (generalized assessments often benchmarked in comparison to others being evaluated or to raise red flags for needed intervention) and summative evaluation (an evaluation of achieved knowledge and skills in a specific area as an end-point to demonstrate academic or clinical qualification at one point in time).

In a context that is pursuing clear and accountable clinical competencies, we must be aware of exactly what feedback is, the globally recognized testing competencies that exist for those who do this work [5], and the nature of optimal learning experiences in resident-education. The International Test Commission (ITC) guideline document was created to inform the creation and use of any assessment procedure that is used in situations where the assessment of people has a serious and meaningful intent, and which, if misused, may result in personal loss or psychological distress. Assessment procedures aligned to competency-based residency programs for professional designation, autonomy, and promotion fit these criteria. The ITC guideline outlines the need for carefully controlled administration with standardized and systematic scoring protocols of expected normal behaviors in education assessment. The closest-aligned evidence-based assessment practice in medical education research is standardized assessments for medical simulations. Simulation is a standardized and evidence-based approach to educating and evaluating residents in rare clinical situations [4]. There is evidence describing that one of the most useful aspects of simulation is the standardized feedback, or debriefing given afterwards [6]. What has not yet been teased out is the extent to which efficacy comes from receiving formative feedback; the implementation of standardized tools, or both. Literature has yet to ask the primary stakeholder of residency programs about what is most supportive of learning – the residents themselves.

**Fig. 1:**
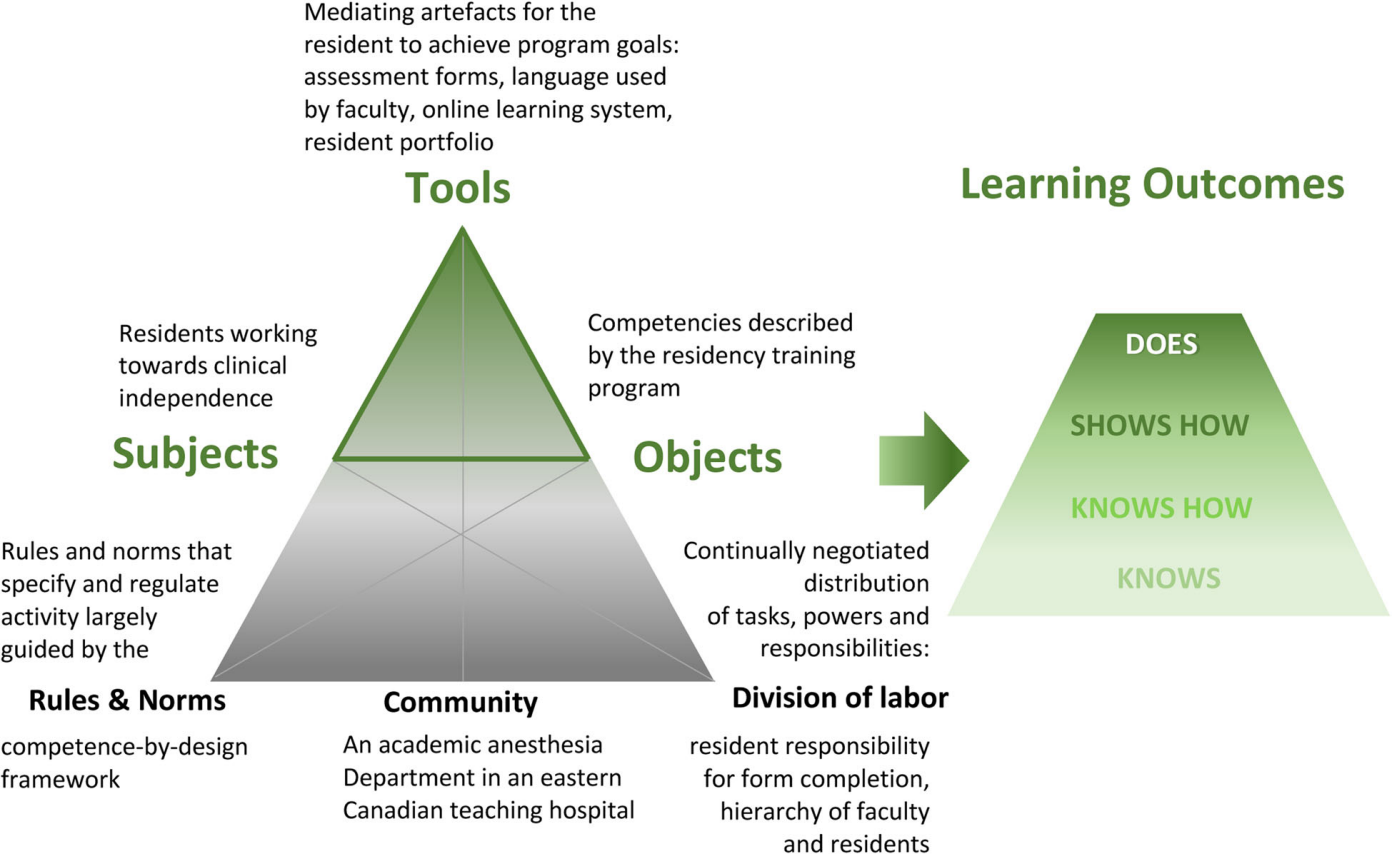
Cultural Historical Activity Theory (CHAT) framework and the Miller’s Pyramid of Clinical Competence. The role of feedback is highlighted as mediating the residents’ interactions and activities towards increasing level of competence. Based on the work in Engestrom, Y. Learning by expanding: An activity-theoretical approach to developmental research. 2nd Ed. Helsinki: Orienta-Konsultit; 1987. and Miller GE. Assessment of clinical skills/competence/performance. Acad Med 1990;9:63–67

From a social constructivist perspective, learning, and human development more broadly, occurs through scaffolding processes. Learning is a staged process of moving from being able to do something with assistance from a more knowledgeable person (anesthesia faculty in this context) to being able to act independently and move from the role of resident to the roles of clinician and educator as a physician [7]. Tools are critical mediating artefacts in this interpersonal exchange and can take many forms. Tools can be symbols, such as language in conversation, and physical documents, such as formal assessment tools and online platforms. One branch of constructivism that helps understand the structure of learning as mediated interpersonal processes is Cultural Historical Activity Theory (CHAT) [8, 9]. CHAT explains the inextricable links between tools as mediating artefacts that are interpreted and contextualized within professional cultures and divisions of labor (Fig. 1). These complex structures of socially situated activity are generally goal-directed, in this case as set out by CBD and standards for professional practice. An important feature of CHAT is the delineation between intended objects of activity (goals) and actual outcomes. It is critical to recognize that goals and outcomes are not always aligned, and that the understanding of and work toward objects of activity are shaping the lived outcomes, in this case resident-learning, skill development, and social-emotional experiences of the resident. CHAT offers a framework for understanding that both context and tools must be considered in the pursuit of studying learning. Individual transformation occurs in social contexts, and in parallel, individual engagement in learning shapes culture [10, 11].

### Research question

Complex social systems have been constructed to ensure assessments and evaluations are made based on our current understanding of their role and function in learning. As recipients and beneficiaries of these practices, the residents’ experiences are central to the meaning and the knowledge constructed out of them and have not been described in the context of medical education. Within this context, we conducted a qualitative study using interpretive phenomenological analysis (IPA) to explore the following questions: (1) What are anesthesia residents’ experiences of feedback in clinical settings; and (2) what are their views on how feedback can help or hinder their progress as learners? [12]

## Methods

Under the constructivist philosophical paradigm, the framework of cultural historical activity theory reveals some of the complexity and highlights primarily the interaction between the faculty and the resident. This interaction is mediated by historically rooted, culturally normed tools – in this situation, the assessment tools and vernacular used by the faculty when providing feedback. Concerned with residents’ perspectives and faced with the lack of published literature on the topic, we engaged in IPA to explore the essence of residents’ experiences of feedback as it impacts learning. IPA is well suited to this task as it seeks to find meaning in the subjective descriptions of individuals’ experiences [13]. Participants were supported to make the implicit nuances of their personal experiences explicit through peer-conversation that unfolded in the focus group sessions. The focus group format, though debated in IPA broadly, was chosen in this study to support the residents to talk explicitly to peers about the details of their experiences. In this way, their conversation with each other became the prompts to provide more detail and subjective feelings and interpretations. This study was approved by the institutional research ethics board, consistent with Canada’s Tri-Council Policy Statement (II) on research with humans.

### Participants

All post-graduate year (PGY) 2 to 5 residents in one anesthesia residency training program who were available to participate in two focus groups were enrolled into the study (*N* = 14). Each resident would have at least 3 years of undergraduate university education prior to training as a medical doctor. Many residents would have a graduate degree (Masters, PhD) by this point in their training, thus having spent between 7 and 12 years in post-secondary education.

### Educational setting

Participating residents were enrolled in a mid-size (25–36 trainees) post-graduate training program in anesthesiology, accredited by the Royal College of Physicians and Surgeons of Canada (RCPSC). The five-year program consists of junior anesthesia and off-service (medicine, surgery, emergency medicine, and obstetrics) rotations in the first year, with senior anesthesia subspecialty and off-service rotations interspersed over the last 3 years. The training objectives of the program are founded on CanMEDS (trademark) framework that describes the seven competencies built into the RCPSC accreditation standard: medical expert, communicator, collaborator, leader, health advocate, scholar and professional. The residency program has strong administrative and educational support, including initiatives such as curriculum mapping and development of entrustable professional activities. All supervising anesthesiologists (“faculty”) have academic appointment, and many are directly involved in educational activities.

At the time of the study, formal resident evaluation practices were conducted through an online platform (one45). Residents were required to submit to their clinical supervisor a daily feedback form based on an entrustability scale [14] and narrative comments to “what the resident did well” and “how could the resident improve”. This form provides the majority of the record of residents’ workplace-based performance. The daily assessments are summated by the faculty in charge of specific rotation into a summative pass/fail evaluation known as In-Training Evaluation Report (ITER) and forwarded to the residency program office. Performance during off-service rotations are assessed on weekly feedback forms specific to the service. A review of assessment forms in the context of advising the resident on their overall progress is undertaken by assigned academic supervisors with whom residents meets quarterly. Since the completion of our study, the program successfully transitioned to CBD. A part of the process of building an evidence-based CBD program included trials of standardized assessment tools and discussions about how to provide feedback and assess competencies required by CBD.

Implementing one of the most useful aspects of medical simulation, structured and standardized assessment tools, might allow residents in routine clinical scenarios to be routinely and formatively evaluated. A team of clinical mentors, AM, AS and RB, thought that by engaging faculty in training and using structured tools, residents could be more consistently assessed on key competencies required for the new CBD residency programs in ways that promote reflection and demonstrate clinical skills in-situ.

The Anesthetists’ Non-Technical Skills (ANTS) is a validated standardized assessment tool, a behavioral marking system, that was created as a framework to guide teachers in their assessment and feedback of learners’ non-technical skills such as teamwork, situational awareness, task management and decision making [15]. Since 2007, the system has been used for both self and peer reflection, to discuss how adverse events were affected by non-technical skills during morbidity and mortality meetings and for formative assessment [16]. It has a positive impact on non-technical skills when used in debriefing and to determine the effects of simulation training [16–21].

The Direct Observation of Procedural Skills (DOPS) is a validated global rating scale used for mandatory formative assessment of residents in the first 2 years of postgraduate training [21–24]. Learners find DOPS highly impactful for their learning, as it helps provide immediate feedback from teachers, stimulation of self-reflection, and targeting of weaknesses [23].

In this context, three investigators (RG, AM and AS) learned to use the standardized tools by studying the ANTS handbook [15] and spending time with a simulation training. An online training module on giving structured feedback using these tools was created by the investigators and completed by all supervising faculty. They used the tools to provide feedback to trainees on the basis of observing their performance during at least 1 day of non-subspecialty, elective cases. ANTS is anesthesia specific and was not modified. DOPS is not anesthesia specific, so the ANZCA 2012 version was adopted [25].

### Focus group sessions

IPA relies for data collection overwhelmingly on individuals’ voices. Residents shared their lived experiences as a group of peers who interacted, challenged, and built on each other’s thoughts. They provided especially rich descriptions of their experiences in focus groups format. Suggested IPA modifications for focus group format were used [26]. All focus groups were moderated by the same medical student who underwent focus group moderation training. Moderation by a junior medical student allowed for a sincere discussion amongst residents, avoiding a power dynamic that would be present if a senior faculty physician led the focus groups. This group dynamic facilitated open and trusting conversation about the topic at hand, sharing specific stories and asking each other questions. The moderator used pre-determined open-ended questions to elicit views on several aspects of feedback: the setting, preparation, aspects of performance reviewed, timing (immediate vs. delayed), contextual information (complexity of cases, overnight call shifts, level of training), clarity of language, appropriateness and relevance, spirit in which it was given, guidance for further improvement, and overall effectiveness.

A second round of focus group conversations hosted approximately 3 months later, asked residents to further reflect on their experiences receiving feedback in the operating room. These focus groups occurred after residents experienced faculty using one of the structured feedback tools as a mediating formative tool to structure feedback conversations initiated by faculty. This gave residents an opportunity to reflect on a new way of getting feedback, mediated by use of feedback tools. Residents also spoke about themes that emerged from the previous discussions and expressed thoughts or feelings that were unaddressed in the previous focus group. One personal interview with one resident was done due to scheduling conflicts. The question script in the focus groups and personal interview remained the same and was reflexive to the conversation that emerged between the residents.

### Data collection and analysis

All focus groups were audio recorded and transcribed by a professional service. The transcripts were reviewed for accuracy and unclear data identified. The moderator field notes and reflections on the transcribed data were attached to provide context. The analytical procedure was reviewed among investigators. A discussion log and an audit trail were maintained by researchers to make clear their depth of understanding of data and share meanings with each other [27].

Gathering of the narrative data through focus groups was followed by qualitative analysis focusing on the experience and the meaning of feedback as a phenomenon central to residents’ learning processes [28, 29]. We aimed to uncover themes emerging from discussions and paint an in-depth picture of the residents’ experiences and understanding from a social constructivist perspective.

Researchers reflected on their own views and biases. Investigators tasked with data analysis (AS, a faculty and a medical student independently), immersed themselves in the data by reading, understanding the transcripts and listening to the original recordings where necessary. Within group interactions, such as points of consensus and disagreement were noted. Transcript analysis and coding was performed using MAXQDA 12 and MAXQDA 2020 (VERBI software) to identify initial themes from the perspectives of both a faculty and student analyst. Each individual participant as well as different focus groups were considered in relation to the overarching themes. This process involved discussion with KR, an educational psychologist, to build a shared understanding of the influence of both the culture of residency training and the theory of learning as a socially constructed process and ensure the credibility of the sensemaking by the investigators. KR then reviewed all the raw data and synthesized the themes from AS and the medical student to report through a narrative format from a social constructivist perspective. A table with multiple verbatim quotes as aligned to ITC standards is provided to facilitate transparency, maintenance of the residents’ voices and serve as a form of triangulation [30].

## Results

The residents were eager to share their experiences and the groups were balanced in terms of participation. All themes but those specific to structured feedback tools were identified from analysis of the first round of focus groups. The second round of focus groups supported the established themes as did new experiences with faculty using structured tools during feedback. After identifying the initial units of meaning, we clustered themes under broad categories of (1) emotional experience, (2) experience of individual faculty, and of (2) specific tools used.

Overall, feedback was felt to be highly impactful for learning and developing professional identity. Residents reported shame and a sense of failure as a result of unfavorable feedback. Residents’ personal interpretations of shame was a topic of significant disagreement. If the feedback was perceived as accurate and delivered constructively by a respected and liked faculty, shame was a strong motivator for learning and improvement. Faculty who were seen by residents to continually re-evaluate and update their practice were respected and their feedback was valued, even when negative. There was resistance to accepting negative feedback from faculty perceived as low performers in those same areas; these experiences were demotivating.

**Fig. 2:**
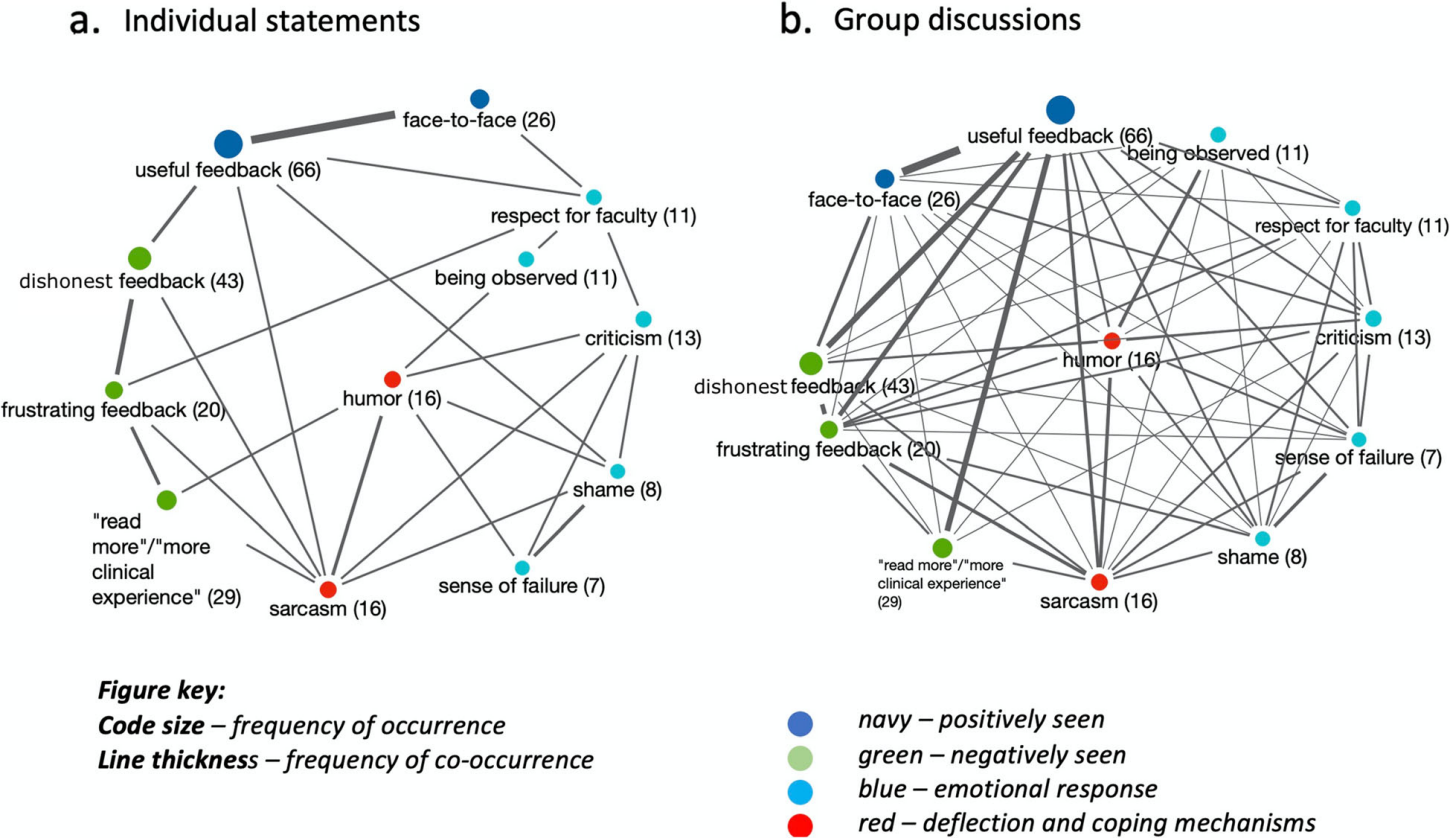
Emotional valence and meaning of feedback - code-proximity maps of thematic co-occurrence (individual/group analysis). Feedback that was seen by residents as inaccurate was perceived as unfair and was often disregarded. Residents’ respect for the faculty providing feedback influenced the meaning – a highly respected faculty giving critical feedback could evoke a sense of shame and failure, however this could in parallel be highly motivating if the feedback was actionable. Criticism from faculty who are perceived as rigid, “picky” and unfair, was universally demoralizing and often dismissed. Humor was used often when poor feedback skills (or routine avoidance of feedback) were centered on faculty (“read more”). Sarcasm was common when these poor skills resulted in feedback that was perceived as personally unfair or unjust. These occurrences gave rise to a sense of resentment. In both individual comments and group discussions, face to face feedback was universally perceived as most useful. Residents felt that it necessitated being observed, although these comments were often accompanied by use of humor, suggesting a mild level of discomfort. Discomfort can be psychologically healthy; it indicates motivation to change, which is a necessary component of learning. In individual and group comments, dishonest feedback was met with sarcasm. Group discussion provided compare-and-contrast discussion (increased frequency and wider linking of co-occurrence), with residents expressing humor and sarcasm on behalf of their peers. Analysis performed using MAXQDA 2020. Berlin: VERBI Software, 2019

Humor was used extensively by individuals and in groups during discomforting conversations. Residents used sarcasm, often on behalf of their peers, while venting frustrations due to feedback that was perceived as inaccurate or unfair to them personally. The meaning of these comments often revealed feelings of resentment and helplessness, especially when such feedback was in written form (Fig. 2).

### Experiences with specific feedback tools

Four types of feedback that vary in their formality, accuracy, relevance, and temporal proximity to clinical experience were described: (1) in situ, (2) daily, (3) 7-day post-clinic written evaluations, and (4) quarterly meetings with academic advisors. The quality of feedback across these four, as described below, was highly dependent on the supervising faculty. Residents believe that much of this faculty-variability is simply due to willingness to put in the effort to do teaching and evaluation work. With that, the residents thought that some professional development on assessment, specifically in situ formative feedback, and its link to learning and gaining clinical expertise can create an ‘aha’ moment for some of the faculty who do not currently give effective evaluations. The residents’ understanding of effective feedback was consistent with evidence-based ITC guidelines for testing, which makes clear the alignment between residents’ understanding of what works for their learning and internationally used guidelines (Table 1).

**Table 1:** International Test Commission Guidelines and Resident Feedback Regarding Evaluation^a^

Theme	ITC Guideline Recommendation	Resident Perspective on What Works
1	Competencies of those administering assessments	
1.1	Professional and ethical standards that affect the way in which the process of testing is carried out and the way in which test users interact with others involved in the process.	“Some are chatty and some are not. Some will sort of teach intermittently as they do it, and some will not. And some are more invested in the structure, and some are not.” “I don’t even think we got to the structured thing. But I wouldn’t say that’s not for lack of trying or out of interest on the part of either me or the staff. Like he was into it. Pulled it up and was like, “Okay, I’ve got this thing. We’ve got to go through it.” *The way in which the staff (test user) engaged the resident in the assessment was not professional. If a task-completion orientation is taken by the person conducting the assessment, then the resident is going to follow that example and know that this is just a thing to complete, not an important part of the learning process.*
1.2	Knowledge of and respect for the rights of the test taker.	“There’s a lot of evaluation fatigue, I think. Because literally every single day we get at least one. And then we have Wednesday and we get 3. And then we get all these other ones on top of it.” “I think over time people will just get fatigued with it and the value that we get out of these will just slowly start to wane. We are already seeing that it can be very useful but it can also be very short and quick and it can also be done in that sort of haphazard manner.” *Residents are not a part of the program design and implementation, leading to forms and processes that might work for a department but is not sensitive to the learning needs of learners.*
1.3	Knowledge of basic psychometric principles and procedures and the technical requirements of tests (e.g. reliability, validity, standardization)	“I think that this is super staff dependent. Some staff can very efficiently sort of run through things, any salient points. And other staff kind of will get to a point and then pontificate a little bit, and use it as a teachable moment and a bit of a discussion point. So I mean it kind of depends on the staff. Because it’s a staff-led sort of feedback model, it depends on them and what they’re going to do with it.” *This comment raises concerns about threats to reliability due to inconsistent use.*
1.4	Knowledge of the specific requirements and processes of the testing tools relevant to one’s area of practice. This includes relevant activities of test administration, reporting, and the provision of feedback to those being assessed.	“The quality is highly variable, I think, depending on the staff and depending on the day. And even with that, some staff are good at it, some staff aren’t. And some staff don’t do it all.” “I would say 100% give you some sense of how you’re doing, just throughout the day. Maybe 90% fill out the forms online. And the number that sit down with you at the end of the day are maybe 20%. That would actually take you aside and sit down with you and talk to you about your performance.” “I think your study is going to come down to it’s staff dependent. Some will solicit your feedback [as a learner] and say, “How can I be a better teacher?” And some, it’s really a one-way sort of thing.” “Some people are good at giving evaluations and it does not matter what the form is, it will be valuable. Some people are just not good at it or don’t care. You can make them do it but it’s not going to add value. Or someone might be very good at giving verbal feedback and not so good at the written feedback. But, at the end of the day, the only thing the program has on record is the written feedback. So, I think there are some limitations, but that’s kind of inherent when you work with 80 different staff.” *Although teaching and assessment of trainees’ skills are competencies, assessment is not explicitly taught or evaluated. This results in highly variable knowledge and skills.*
1.5	Oral, written and interpersonal communication skills sufficient for the preparation of test takers, administration of tests, and the provision of feedback of test results.	“It wasn’t too short, it wasn’t too long. We had a great day, a standard day, got done one time, took the full half hour, not in the OR, reviewed in this (private) room. It was good. You know, it was nice structured formal feedback. There were very good points and observations discussed.” “Some staff appreciate that there’s variability between the practices. Some staff even if they acknowledge it still chastise you sometimes in a negative way if you don’t do something how they do it. So that can sometimes be frustrating and sort of mar any kind of other feedback you’re going to get from them that day because you know it is stupid and annoying and you can’t do anything about it, I find.” *The interpersonal skills of the staff are not sufficient in this reported experience. Feedback must be about valid observations of the competencies being developed, not the preferred habits of a specific staff. Further. To report being “chastised in a negative way” indicates that the feedback was not delivered in an effective manner.*
1.6	Conduct communications with due concern for the sensitivities of the person being assessed and other relevant parties.	M: “On the form we currently get on [the online system], there’s a thing to click if you’ve had a chance to discuss this with your preceptor. I always just click yes, even though pretty much I never. You know, a lot of days you don’t actually even really have any kind of formal feedback other than just like “Good job today. See ya later.” T: “I click whatever they clicked” M: “Me too” (laughs) T: “even if they’re like “we met” I’m like, “Yeah, sure we met” M: “I agree with you” T: “Yeah. Or we didn’t and like, “you did say something, but whatever”. *It is important to acknowledge the power dynamic of the preceptor over the resident. An online tool providing an option to disagree with the reporting of a preceptor is unlikely to produce results because the residents do not feel they are in a position to disagree.* “We did it [formal private conversation] in the OR. ..they (*assessor*) were like “do you care?”…[I said] Everybody just watched me do all the things you’re going to talk about to me right now. So…” *It is not considerate of the potential sensitivities of the residents’ needs to ask in front of other people to give the feedback in front of other people when the standardized assessment tool indicates to take a small amount of time in a private space. Given power dynamics, it is unlikely that a resident would say they want the standard private time if the senior staff conducting the assessment is indicating they do not want to.*
1.8	Knowing when and when not to use tests.	“It was complicated to the point of not possible on our day. We just had a busier day. And all day we were kind of like, oh, we’ve got to try to find time for this, maybe we’ll fit it in here, there. Oh, we’re not supposed to do it in the OR. And then, in the end, kind of like half did it in the OR because it was a busy day. I don’t think I even got the structured thing.” “I had the opposite experience. I was on OBs in a gynae room with like 3 cases and a fast surgeon. So we just took our time. My staff was in the corner with a folder, doing the assessment, watching me. And we literally at the end of the day could like …because we finished at like 3 pm, just went and found a room and did the whole structured feedback. There was tons of time to do it. But I can totally see if you have a busy day and lots of things are going on that it would be very difficult to do.” *Timing is a logistical constraint for these assessments. Setting up a system that does not force assessments when the time is not available would improve the quality of assessments when there is appropriate time to complete them.*
1.9	Choice and evaluation of alternative tests	“I think that there are days where the ANTS part is more important and days where the observational part is more important. …Like for the stuff we don’t do a tonne of, like weird blocks or fibre optics or thoracic epidurals, or whatever it might be, those are days that [fit the ANTS] better. Having the structured feedback for those is pretty important.” *We trialed two standardized feedback forms. Having choice was contextually appropriate from the residents’ perspectives.*
1.10	Knowledge, understanding and skills relating to the process of testing: What test users need to be able to do to administer, score, and interpret tests.	“If we are talking about quality, it’s not only a question of whether or not they fill it out but also what they put in there. So some stuff will go on no matter what, just fill it out like in a row, kind of wherever they think you fit in. Whereas some staff are very thoughtful and you can tell they put a lot of effort into it to give you specific feedback of things you can actually work on. And then some other staff will say “keep reading”, which we see millions times.” *Test users are critical to the process and residents see a high degree of variability. This influences their learning.*
1.11	Report writing and feedback mechanisms that are accurate, timely, consistent and useful. Include within written reports a clear summary, and when relevant, specific recommendations.	“The feedback that’s most useful, when it was really good, usually revolves around decision-making and where we can identify points where critical decisions had to be made, the alternatives, and then giving me feedback about what can be done next time. That becomes more useful in the grand scheme of things.” “Most staff, I would say, make an effort to make at least some sort of acknowledgement of how the day went… if there was an issue, that would be brought up at that time. Whether that gets translated in terms of written output …it takes a big steep drop-off after that. Because I think some people really sort of substitute what they’ve talked about as more meaningful and not really necessary to, you know, do the written thing if you’ve discussed it.” “I’d say the minority would actually at the end of the day like bring you into the lounge or somewhere and sit you down, and actually do like a ‘what you did well’. Like, try to be more structured. Less than 10% actually. The vast majority are like ‘See ya!’”. “The staff that give the more high quality feedback, it’s gotten really useful and the feedback prompts very good reflection that I then carry over to, you know, the rest of rotation. And some I’ve gotten is completely useless, and also sometimes doesn’t match between the written and oral. So at the end of the day, they’ll say “Good job, everything went well today, no issues.” And then I get the written form back and it says “I had to be in the room for technical skills.” You know, it totally doesn’t match my perception and it doesn’t match what they said. It is not reflective of me and the actual experience we had in the OR.” *The structure and forms are conducive to accurate, timely, consistent and useful feedback, though whether this happens is variable depending on the assessor’s knowledge, attitudes and skills.*
2	Characteristics of Standardized Assessment Tools and Procedures	
2.1	Supported by evidence of reliability and validity of their intended purpose.	“I think in terms of the timeliness and the face-to-face components of receiving feedback, I think these standardized tools ensured that that actually happened because there was something more structured that we both had to pay attention to. It was more than just like a “Good day. See ya.” So I think it did impact that significantly. All of us, even if it was only 5 min, had a point in time where we knew we were getting feedback and our preceptor knew they were giving us feedback, and it was happening on the day, face-to-face.” *Having a standardized form introduced increased the consistency of feedback, which is an indication of reliability.*
2.2.	The assessment procedure provides evidence to support the inferences that may be drawn from the test.	“I think it’s a general consensus that some days are good and some days are bad. And for me, the way that you address that is you have a cumulative number of experiences with a certain staff. And at the end of a time period, you have a more encompassing thing when you have some time to deal with it.” “I think that because everyone is going through a structuring thing, you at least have a face-to-face time to discuss any issue that might have come up. So think you aren’t going to run into what [participant] was talking about with regard to having a brief discussion of the day and then getting an eval online that’s like “What?!” Because there were specific times where you’re going through the thing, and the staff would say, “I thought you did this really well” or whatever “but maybe you were a little bit … I don’t know what happened here” And you could be like, “Oh, that was because…”. And then it was discussed and it was sort of like nothing could be hidden with that because it’s face-to-face. So I think as long as you’re following some sort of structure and you have a face-to-face discussion about it, the [verbal and written feedback will be reliable]. “We have to do quarterly… There are supposed to be reviews with our academic mentor. And [the forms submitted online] is the information that our academic mentor has about us. They read most of or all of the feedback forms that we get on these daily things. We have these meetings 4 times a year and we discuss them. So my mentor is like super on top of meeting 4 times a year. She goes through and she literally picks out things that people have written. So if it’s an informative evaluation from a given day, I would say that is useful for her because that’s how she’s evaluating me and like doing her quarterly review of how I’m doing.” *Overall the program has corrections against specific inaccurate forms because a collective of forms, in tandem with a mentorship relationship, is used quarterly to support resident progress.*
2.3	Logistically feasible within and related to the test setting.	“I think day-to-day, to rely on the fact that you’re going to have half an hour to sit down and talk about something is unreliable.” “The verbal feedback that’s given on the day of, it’s much more specific, it’s more precise, it’s more relevant, and a lot more useful.” *There was a range of scenarios discussed – when logistically feasible, the evaluations are effective. When not logistically feasible, it feels forced, does not contribute to learning and leads to evaluation fatigue.*

^a^Resident’s verbatim words during the focus group are in quotes. Paraphrased words from the residents’ verbatim quotes are in []. Researchers’ connections to ITC guidelines are in *italics*

The ITC guidelines outline what a quality assessment tool is, as well as the knowledge, skills, abilities and other personal characteristics requisite of those conducting evaluations of others that have consequences for the test-takers’ work or personal lives. The guidelines clarify that these standards apply beyond what might be formally termed a “test” to any assessment procedure that provides estimates of performance and involve the drawing of inferences from samples of behavior in professional practice settings where there are substantial consequences to the person being assessed, such as medical accreditations and career progression

Point-of-care feedback in clinical settings as a procedure is taking place, or immediately after an event warranting feedback, was consistently described by residents as specific, precise, and useful or actionable. Residents consistently described that these moments helped them to learn and improve in tangible ways. Residents welcomed this feedback, including negative feedback, because it was the most accurate and valuable evaluations they receive. Residents explained that this is a norm for some faculty, while others never give point-of-care feedback. To illustrate the high value placed on point-of-care feedback, they expressed regret for not receiving feedback on their management of out of operating room emergencies while on call and without direct supervision by anesthesia faculty. It was during these cases that residents felt they did their “best stuff”, but also felt they would stand to learn the most from point-of-care formative feedback.

The weekly written feedback was non-specific. This non-specificity and sometimes inaccurate assessment were thought to be due to recall error. Residents shared they occasionally had written feedback about clinics that they had never actually worked in before. This is a clear example of recall error as time lags between the resident performance and the faculty assessment. This assessment fails to meet standards of accuracy, cannot be used as evaluation against learning targets, and provides no feedback facilitative of resident-learning.

The fourth type of evaluation described was the quarterly meetings with an academic mentor. These meetings were useful because the mentor could help the resident see trends in comments provided in submitted ITER forms over time, set goals and be reflective on one’s practice. This was most useful when the ITER forms had specific and accurate descriptions of work and areas needing improvement. For the scale of evaluation (overall competence aligned to program standards as framed by CBD), residents saw these meetings as accurate, relevant and useful for continued learning, goal setting, and reflective practice.

### Timeliness

The daily evaluation forms were explained as being highly variable in usefulness based on when the feedback was completed. Some faculty completed a form while still in the clinical context, others completed by the end of the day, others within 7 days while some never submitted the forms. Though quality feedback (defined by residents as specific and actionable) was most often timely, some faculty completed the forms diligently but still gave only generic comments about reading and practicing more in non-specified ways.

### Usefulness

The most resounding comment made, multiple times across residents, was how useless and demoralizing it was for a faculty to be vague by writing “read more” or “continue to gain experience”. Residents saw no learning benefit from these comments that are received often: “they are not useful and at times are harmful to a culture of learning”. Residents also described the ways in which forms were useful. The most useful part of the forms was the open-ended text responses where faculty could be specific. Residents shared that they much preferred the open-ended responses over the entrustablility scale ratings of excellence. While the overall progression of skill along the scale was understood, they felt when used in the operating room setting, the scale could be confusing as “I did not have to be there” was a response option that could be selected by a faculty who was in the operating room with them all day, as well as the one who was not there at all. They shared that moving from scale scores to open ended qualitative feedback shifted the perspective of the residents from being achievement oriented (“seeking the 5 out of 5”) to being improvement oriented ( “… given where I am now, what is the next thing I need to work on to continue my learning?”).

### Shared responsibility

A positive story about form completion and timely specific feedback focused on a faculty who filled out the form in advance of meeting with a resident in a quiet space, discussed the feedback given and before submitting asked the resident if they thought the evaluation was accurate. This opened the line of communication and fostered a shared responsibility for assessment and learning, using the form as a meaningfully situated mediating artefact. Residents welcomed the idea of increased responsibility and voice in their assessments but worried that this might sometimes not be welcome by faculty who see teaching “as a one-way sort of thing”. On occasion residents have experienced faculty asking them what more they need or how they as faculty can be better teachers for the residents. Though this was greatly appreciated, and residents desired more shared responsibility, it was a rare experience. Residents explained that they spend considerable time ensuring that feedback forms are completed and brainstormed about how they could take on more responsibility by being strategic in how they seek feedback. Knowing that they appreciated specific actionable constructive criticism, the group discussed not just asking to complete a form or “how did I do today?” but instead to ask specific questions that they are thinking about relative to specific competencies and want insight on. Residents also shared the idea that they could ask faculty at the beginning of the day for targeted feedback. Because there are so many competencies to meet, and not all of them are equally relevant across contexts and days, the residents thought they could ask faculty to specifically watch out for a specific skill on a given day rather than trying to “tackle everything all of the time”.

### Feedback “burnout”

The residents expressed that they often had more than one assessment form within 1 day. These forms were a source of frustration, as the residents felt pressure from the program to prompt faculty for their completion. The expectation was there even when residents knew they were emailing a faculty who had never filled the form out before and was unlikely to start doing so. This is an example of the form being forced when it is not functioning as a mediating tool in support of formative feedback from faculty. This put residents in a vulnerable position. Both program administrators and attending faculty are in positions of power relative to the residents’ career progression. The residents felt they had to balance not wanting to fill an inbox of an attending faculty who will not complete the form, against the need to send a request to fill program requirements. Residents perceived a clear distinction between form completion that fulfills the accreditation needs of the program and form usage that serves their needs as learners and faculty-needs as teachers. For example, the daily evaluation interval was insufficiently long for the resident to demonstrate feedback-based improvement when working with a different faculty every day. However, they were still under the expectation to send daily forms to the same faculty for each of the several consecutive days of work when one evaluation would better capture their progress and obviate repetition. The residents felt that the overall trend in the program preparing for transition to CBD was toward increased quantity, but not necessarily quality, of feedback and evaluation.

### Standardized tools in clinical learning contexts as mediating artefacts

The DOPS was seen as a useful tool in highly specialized clinical contexts where there were specific procedural skills that could be better refined over time. Examples where the DOPS was effective for residents were well defined procedures such as invasive vascular access in cardiac anesthesia and lung separation in thoracic anesthesia contexts. They also noted that some supervisors need explicit instruction on how to give feedback, with or without standardized tools.

The most frequently cited benefit of ANTS-guided feedback was that it necessitated direct, face-to-face discussion. The specific nature of feedback was welcome, especially if the day was eventful and included complicated, challenging cases. It was felt to contribute little to evaluation at the end of a routine day, although the framework and the vernacular it provided could be helpful for those faculty who struggled with giving feedback. For these tools to be useful and not lead to assessment-burnout, residents thought that a daily tool with fewer items that takes fewer than 10 min would be ideal. A tick-box at the top of the form could indicate if the day was too busy to get to the task. This would set up expectation and logistic feasibility to use the tool almost daily. The tick-box opting out of given clinical circumstances was described as more accountable and trackable than supervisors simply not completing a form and residents left wondering if they should follow up to ask for the program-required feedback. Ten minutes, as opposed to the typical 30 min the feedback process required, was a way to reduce assessment-fatigue and increase commitment and habit of doing these reflections at the end of a day. Alternatively, an idea was shared that ANTS could be initiated by a resident following more remarkable cases. The residents saw that the forms could help divert from the sometimes “haphazard manner” in which they receive feedback, though they also cautioned that a new form is insufficient if the supervisor does not know how to observe, assess the skills, and give timely and specific feedback that the resident can act on. Need for coordination of all assessment types across the program was important to residents. The need for formative evaluation that promoted learning was embraced, but assessment that distracted from their professional development or was not done well by the supervisor was demoralizing. Critical to the success of the tools was the supervisor’s ability to fluidly use the tool in a concise manner as relevant to what happened and what could improve. Residents lost focus when supervisors were talking about the tool itself or trying to remember how to use it. It was felt that to avert this awkwardness and to increase relevance, the entire department must embrace a tool and ensure sufficient capacity development in its use before it is implemented. In other words, the tool could be effective if meaningfully integrated into the activity systems that structure residency programs. This onboarding across faculty would make clear that the object of activity was resident learning as mediated by use of a form, and not form completion as an end-goal.

## Discussion

This research presents the first account of anesthesia residents’ experiences with in-training feedback. In addition to providing additional insights into how we may go about feedback to promote learning, we illustrated and aligned the residents’ observations about feedback with the most general of guidelines on testing such as are provided by ITC (Table 1).

Potential limitations of this research include a sample that was confined to a single residency program. In addition, while we used modifications for the focus group approach which offered certain benefits, we may have failed to elicit some of the more personal psychological and idiographic dimensions unlikely to be shared in that setting. Further research is needed to address the relative affordances and constraints of focus groups in IPA.

Observations about their daily performance are experienced by residents as deeply impactful and personal. Feedback received for sub-par performance was appreciated when delivered in a constructive way. Conversely, there was an agreement that seemingly innocuous generic statements such as “needs to read more” or “needs more experience” were among the most harmful. It is clear that feedback plays an important role in personal and educational experience even at the final stretch of a long educational path.

By successfully attaining the post-graduate medical education level, residents have demonstrated that they are expert learners, preparing for a career of life-long-education. This insight is aligned with effective incremental learning and can directly inform how programs go about the CBD’s proposed milestone-oriented assessment structure.

An example of such professional development in an area of clinical practice is the primer document for assessment in Graduate Medical Education, created by the American Board of Pediatrics and The Association of Pediatric Program Directors [31]. This document is too specific to pediatric contexts to be directly applicable to other residency program areas. The ITC guidelines are too general, as they are constructed for global and wide-use for all psychological, professional, and educational testing. The RCPSC Workplace-Based Assessment Implementation Guide introduces the components of trainee assessment in CBME and practical tips on how to operationalize assessments. While techniques for providing formative feedback are clearly described (for example, RX-OCR) the focus is clearly on documenting residents’ progress for the purpose of promotion or remediation. Norcini and Burch (2007) highlighted in their guide to formative assessment the paucity of evidence specific to medical education and emphasized the importance of faculty development given how often clinicians fail to give feedback [32]. In a 2010 review of the impact of formative feedback on medical trainee performance, Miller and Archer found significant variability in the context and facilitation techniques [33]. It is clear from the comments in our focus groups that feedback given is not necessarily feedback received and internalized. There is a need for more research into trainee evaluation practices that are most conducive to learning and skill advancement as well as for development of evidence-based guidelines for formative feedback within clinical settings. Evaluation competency of peers, residents, and other trainees is already within the scope of physician as a scholar, as defined by CanMEDS. It is, however, rarely formally evaluated itself.

The results of our research inform us about the concept of ‘resident-led evaluation’. This concept does not mean that the residents always know what they don’t know – mentors are required to guide the resident and identify knowledge or skill gaps in developing competence [34]. Rather, the residents can become self-regulated learners, integrating through meta-cognitive processes the range of competencies that they are to develop professionally. Programs can also establish norms that expect residents to start a routine clinical engagement by asking an attending faculty to look out for a specific skill that they are wanting to focus on in their learning on a given day. We cannot assess everything at the same time. Standardized forms will not succeed if they involve an extensive checklist of all competencies, and as the residents noted, this will lead to form fatigue for all, with no benefit of accuracy or utility. Rather, sharing responsibility for learning by taking turns between attending faculty and resident to set (or co-establish) the goals for evaluation with timely and actionable feedback for learning is the goal.

## Conclusion

Residents understood their dual role of clinician and learner and had a detailed sense of what promotes their learning. There is a need for all clinicians who evaluate within CBD programs to demonstrate competence as outlined by ITC guidelines. This requires system-level support to develop the competence and understand why it is important. Faculty competencies in evaluation should also be formatively assessed.

### Practice points

- Residents highly value formative feedback and its role in their training
- Useful feedback is seen as timely, open to discussion, and is a result of effortful, skilled observation on part of the preceptor
- Standardized feedback tools are valued as they require face-to-face feedback, but are not seen as a substitute for preceptors’ skill and motivation
- Residents can be self-regulated learners sharing the responsibility for performance goal setting and feedback
- The necessity to fulfill administrative requirements can contribute to burnout when it competes with residents’ ability to obtain useful feedback

## Data Availability

Datasets analyzed during the current study are available from the corresponding author on reasonable request. All potentially identifying information will be removed from the qualitative transcripts before being shared.

## Abbreviations

ITC: International Test Commission
DOPS: The Direct Observation of Procedural Skills
RCPSC: Royal College of Physicians and Surgeons of Canada
CBD: Competence by Design
CBME: Competency-Based Medical Education
PGY: Post-graduate Year
ITER: In-Training Evaluation Report
CHAT: Cultural Historical Activity Theory
ANTS: The Anesthetists’ Non-Technical Skills
IPA: Interpretive Phenomenological Analysis
